# Comparison of the Effects of Tamarind Seed Extract (Tamarindus Indica L.) with Sertraline in Treating Premature Ejaculation: A Randomized Double-blind Trial: Tamarind vs. Sertraline in PE Treatment

**DOI:** 10.31661/gmj.v14i.4015

**Published:** 2025-08-27

**Authors:** Ali Sahraian, Iman Shamohammadi, Somayeh Daneshvar, Payam Sadeghi

**Affiliations:** ^1^ Research Center for Psychiatry and Behavior Science, Shiraz University of Medical Sciences, Shiraz, Iran; ^2^ Department of Urology, Shiraz University of Medical Sciences, Shiraz, Iran

**Keywords:** Premature Ejaculation, Tamarind, Sertraline, Treatment, Trial

## Abstract

**Background:**

Premature ejaculation (PE) is a prevalent male sexual disorder often untreated due to embarrassment. This study compares the efficacy of tamarind seed extract (Tamarindus indica L.) with sertraline in treating PE.

**Materials and Methods:**

In this randomized, double-blind, parallel-group trial, 41 men diagnosed with PE at urology and psychiatric clinics in Shiraz, Iran (2023) were enrolled. Participants were randomly assigned to receive either 80 mg tamarind seed extract capsules (Group A, n=20) or 50 mg sertraline tablets (Group B, n=21) daily for 4 weeks. The primary outcome was the Premature Ejaculation Diagnostic Tool (PEDT) score, with secondary outcomes including International Index of Erectile Function (IIEF) scores (erectile function, orgasmic function, sexual desire, intercourse satisfaction, overall satisfaction) and side effects. Data were analyzed using SPSS version 22 with t-tests and ANOVA (P0.05).

**Results:**

Both treatments significantly improved PEDT scores (P=0.001, η_p^2=0.76, 95% CI for mean difference: [-6.95, -4.95]) and IIEF subscales (P0.01). No significant differences were observed between groups (P=0.69, η_p^2=0.10) or in the time-group interaction (P=0.42, η_p^2=0.15). Side effects were minimal in both groups.

**Conclusion:**

The findings indicate a potential relationship between tamarind seed extract and improvements in premature ejaculation and related factors, comparable to sertraline; however, these results should be interpreted with caution and require further validation through additional research. Trial registration number: IRCT20140926019295N4.

## Introduction

Orgasm is a feature that is perhaps unique to human beings and is a brain process that normally accompanies the ejaculation of the semen. Normal ejaculation is a highly coordinated physiological process involving the release and expulsion phases controlled by the autonomic and somatic nervous systems [1].

Premature Ejaculation (PE) is defined by the American Urological Association as "ejaculation that occurs sooner than desired either before or shortly after penetration, causing distress to either one or both partners"; the International Society of Sexual Medicine (ISSM) adds that it represents a clinically significant reduction in ejaculatory control and capacity [2]. Estimates suggest that PE affects between 1% to 5% of men although some studies indicate that the actual prevalence may be higher [3]. PE can affect various aspects of a person's life, including mental and emotional well-being, as well as interpersonal relationships. Unfortunately, this issue is often overlooked and remains untreated, largely due to low intervention rates and the feelings of shame associated with it [4].

There are both pharmacological and psycho-behavioral treatments available for premature ejaculation. Psycho-behavioral approaches include techniques such as stop-start methods, squeeze techniques, masturbation before intercourse, and even yoga [5][6]. However, these methods are not recommended as first-line treatments for long-term premature ejaculation [7] as they are generally less effective than pharmacological treatments in prolonging the Intravaginal Ejaculation Latency Time (IELT) [8]. Pharmacological treatments can be classified as either topical or oral. Oral medications can be taken continuously or as needed prior to intercourse. Some common topical medications include various forms of lidocaine or prilocaine [9]. Currently, fluoxetine, sertraline, paroxetine, and citalopram are common selective serotonin reuptake inhibitors (SSRIs) used for the treatment of PE [10].

Sertraline is an approved medication for treating premature ejaculation (PE). A meta-analysis by Zhan-Miao [11] reviewed randomized controlled trials from multiple databases and found that sertraline significantly prolonged IELT and improved sexual satisfaction in patients with PE. However, it may also increase the risk of gastrointestinal distress.

Given that drug treatments often come with complications and behavioral therapies are not as effective as pharmacological options, there has been an increase in studies exploring herbal and natural remedies. In recent years, the use of various alternative medicine approaches has grown in many countries. Numerous herbal products have been developed in traditional and alternative medicine for individuals seeking to enhance their sexual lives (7). Some previous studies [12][13][14] have evaluated various herbal products in patients with premature ejaculation, but the findings have been mixed and limited. Consequently, the efficacy of these herbal agents in addressing sexual problems remains unclear.

For instance, in a review by Malviya et al. [15], various medicinal plants were explored for their effects on male sexual disorders. Notable findings showed that plants like Alpinia calcarea, Anchilus pyrethrum, Anthium graveolens, and Asparagus adhesins improved sexual behavior in male rats by enhancing libido, reducing erectile latency and increasing erections and ejaculations. For instance, Anchilus pyrethrum root extract boosted sexual organ weight, behavior, and testosterone levels. Garcinia kola also positively affected male fertility by increasing the sperm count and testosterone. Additionally, saffron (Crocus sativus) and maca (Lepidium meyenii) were noted for improving sexual function, with saffron increasing erection frequency and maca enhancing the semen volume and sperm motility [16].

Tamarind is one of the herbal products that has recently been studied for its potential in treating premature ejaculation (PE). The scientific name for tamarind is Tamarindus indica, and it is a medicinal plant known for its high , anti-inflammatory effects, and [17][18][19]. Various parts of the tamarind plant are utilized in food products, industries, and medicine [20]. Previous studies have reported on the medical uses of tamarind seeds for different purposes [18][21][22]. In Iranian traditional medicine, tamarind seed powder has been recommended for managing premature ejaculation; however, there is currently no evidence-based information commonly used treatments like Sertraline.

In Iran, a study by Homayounfar et al [16] compared tamarind seed extract, paroxetine, and placebo in treating premature ejaculation over 4 weeks. The study found that paroxetine significantly improved intravaginal ejaculation latency time and sexual function compared to tamarind and placebo, with tamarind showing no significant advantage over placebo. These findings highlight the need for further research to evaluate tamarind’s efficacy relative to established treatments.

Given the significance of addressing premature ejaculation and the ongoing search for new treatments, this study explores the potential of tamarind seed extract, which is frequently highlighted in various traditional medicine sources for its benefits. Additionally, tamarind seeds are easily consumed in different parts of the world without complications. The objective of this study was to compare the efficacy and safety of tamarind seed extract (Tamarindus indica L.) with sertraline in treating premature ejaculation, including their effects on sexual function and side effects. sertraline was selected as the comparator in this study due to its established use in PE treatment, supported by evidence of its efficacy in prolonging intravaginal ejaculation latency time and improving sexual satisfaction, as demonstrated in a meta-analysis [11].

## Materials and Methods

### Study Design

This randomized, double-blind, parallel-group superiority trial was designed to compare the efficacy of tamarind seed extract with sertraline in treating premature ejaculation. A sample size of 41 participants (20 tamarind, 21 sertraline) was determined based on a power analysis to detect a significant difference in PEDT scores between groups, assuming a power of 80%, a significance level of 0.05, and an effect size derived from prior studies. Participants were recruited from the urology clinic of Shahid Faghihi Hospital and the Psychiatric clinics of Shahid Motahari, Imam Reza, and Ebnsina Hospitals in Shiraz, Iran, using a permutation block design. The study was conducted from April 2024 to June 2024, with participant recruitment occurring from April 20, 2024, to June 22, 2024, and follow-up assessments completed by June 22, 2024. Trial sites were selected based on the availability of urology and psychiatric clinics; no specific eligibility criteria were defined for sites or interventionists. Before the study, the Institutional Review Board of the Shiraz University of Medical Sciences approved the experimental protocol (ID: IR.SUMS.MED.REC.1402.542). All subjects provided written informed consent. The trial was registered with the Iranian Registry of Clinical Trials (IRCT20140926019295N4, https://irct.behdasht.gov.ir/trial/76268) on 2024-04-09. The trial protocol is available upon request from the principal investigator (Ali Sahraian, sahraian@sums.ac.ir) or via the Ethics Committee of Shiraz University of Medical Sciences. No patients or public were involved in the design, conduct, or reporting of this trial. One change was made to the trial protocol after commencement: the IELT questionnaire was removed due to inadequate patient cooperation in completing the assessment.

### Study Participants and Sample Size

Participants in the study were required to meet the following criteria: being in a monogamous and stable sexual relationship with a female partner for a minimum of 6 months, being engaged in sexual intercourse at least once a week, ejaculating consistently either before or within approximately 2 minutes of vaginal penetration, being between 20 and 50 years, having a score of above 8 on the PEDT questionnaire, and signing the written informed consent. Exclusion criteria included severe side effects deemed dangerous by a doctor, severe erectile dysfunction after treatment, failure to follow instructions, use of outside treatments, chronic use of neuropsychiatric medications or narcotics, and conditions like chronic constipation or ulcerative colitis. Moreover, men who had been taking drugs that could affect their ejaculatory function were also excluded. Recruitment occurred from April 20, 2024, to June 22, 2024, with follow-up assessments conducted from May to June 2024. The trial concluded as planned upon completion of recruitment and follow-up on June 22, 2024.

### Measures

International Index of Erectile Function (IIEF) Questionnaire: This 15-question tool evaluates five areas of sexual health including erectile function, orgasmic function, sexual desire, intercourse satisfaction, and overall satisfaction. Its Persian translation was validated by Pakpour et al. in 2002.

The following questions assess various aspects of sexual function:

- Questions 1, 2, 3, 4, 5, and 15 evaluate erectile function.

- Questions 9 and 10 assess orgasmic function.

- Questions 11 and 12 assess the degree of sexual desire.

- Questions 6, 7, and 8 measure the differences in intercourse satisfaction.

- Questions 13 and 14 determine the overall satisfaction score [23][24].

The Premature Ejaculation Diagnostic Tool (PEDT): This questionnaire consists of 5 questions. A score of 8 or less indicates no premature ejaculation, 9 or 10 suggests probable premature ejaculation, and 11 or more confirms premature ejaculation [25].

### Randomization and Allocation

The project manager assigned the patients to two groups, A and B, using a permutation block design with 8 blocks of 5. The random allocation sequence was generated by a computer-generated random number table using a permutation block design with 8 blocks of 5, managed by the researcher responsible for randomization (Payam Sadeghi). The study maintained blinding at the patient level and for outcome assessors and analysts, ensuring that both tamarind kernel capsules and standard drug capsules were indistinguishable. Allocation was concealed using identical capsules (same shape and packaging), with randomization codes stored securely by the researcher responsible for randomization. Only the researcher responsible for randomization could decode the capsule contents. Group A received 80 mg tamarind seed extract capsules daily, taken orally with water in the morning. Group B received 50 mg sertraline tablets daily, taken orally in the evening to for 4 weeks. No concomitant care or medications were permitted during the trial, as specified by the exclusion criteria. During the first visit, patients completed a demographic form and the PEDT scale and provided an initial history to confirm eligibility.

**Figure-1:**
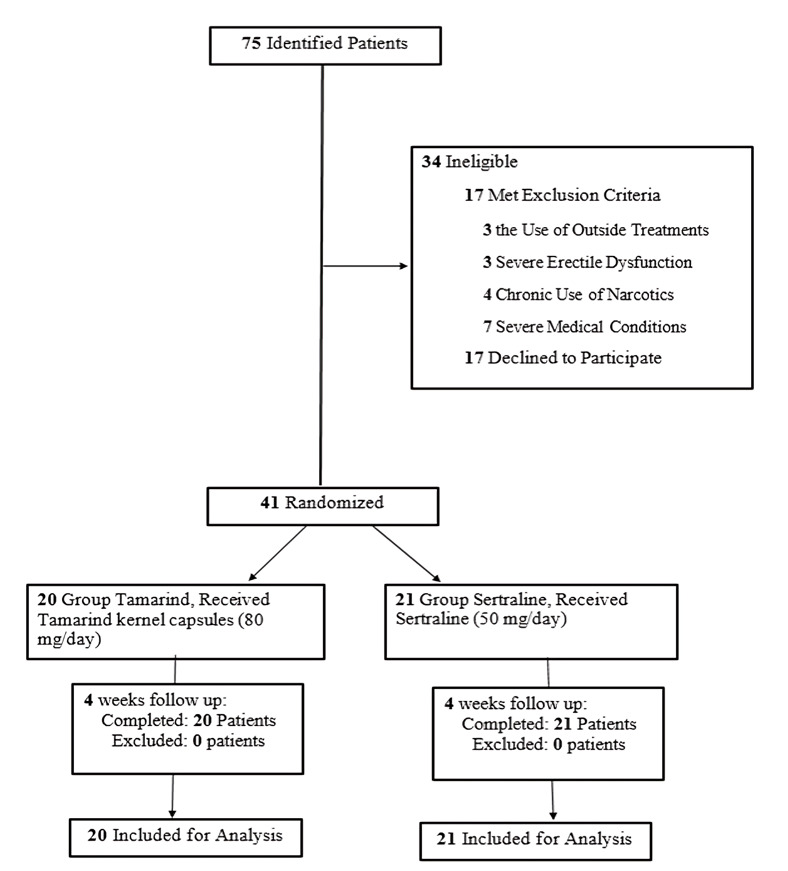
Figure 1. This figure represents the CONSORT flow diagram of the study.

They were assigned a project participation file number and signed a consent form, after which they received and learned how to use the IIEF form. Following random assignment to either group, patients returned for a follow-up visit after 4 weeks, where they discussed the side effects, underwent a general examination, and completed the drug side effects form, IIEF form, and PEDT end-of-treatment form (see Figure-1). No interim analyses or stopping guidelines were planned due to the short 4-week duration of the trial. The primary outcome, as prespecified in the trial protocol, was the PEDT score, measured as the mean score at baseline and after 4 weeks. Secondary outcomes, also prespecified, included International Index of Erectile Function (IIEF) scores across five domains (erectile function, orgasmic function, sexual desire, intercourse satisfaction, and overall satisfaction), assessed as mean scores at baseline and 4 weeks, and side effects, recorded via a standardized form at the 4-week follow-up. Initially, the IELT was planned as a secondary outcome but was removed from the protocol due to inadequate patient cooperation in completing the assessment. Harms were systematically assessed using a standardized side effects form completed at the 4-week follow-up visit, with general examination and clinician oversight for severe adverse events.

### Statistical Analysis

The collected data were entered into SPSS version 22 (IBM, Armonk, New York, United States) software and analyzed statistically. Quantitative data are reported as Mean ± SD, while qualitative data are presented as Number (percentage). The normality of the quantitative data was assessed using the Shapiro-Wilk test, which indicated that the data followed a normal distribution. For analysis, the following statistical tests were employed: Independent Samples t-test, Paired Samples t-test, Wilcoxon Signed Ranks Test, Pearson correlation, Spearman correlation, and Chi-square tests. A significance level of less than 0.05 was considered for all analyses.

## Results

**Table:** Table1. Demographic Information and Statistical Variables at Baseline for Participants

**Variable**	**Tamarind Grup (n=20) **	**Sertraline Grup (n=21) **	P-Value	**Statistical Test**
	**M**± **SD**			
**Patients’ Age (years) **	35.65±1.36	36.81±1.52	0.57	t(39) = 0.56
**Patients’ Wife’s Age (years)**	31.70±1.47	33.24±1.56	0.48	t(39) = 0.72,
Educatinal Attainment (Patient)			0.59	χ²(6, N=41) = 4.63,
**Secndary Edu.**	2	5		
**Diplma**	5	4		
**Bachelr's**	8	9		
**Pstgraduate**	5	3		
Educatinal Attainment (Wife)			0.23	χ²(6, N=41) = 8.06
**Secndary Edu.**	6	9		
**Diplma**	4	4		
**Bachelr's**	7	6		
**Pstgraduate**	3	2		
PEDT			0.95	χ²(1, N=41) = 0.003
**Nt Having PE**	0	0		
**Suspect t PE**	2	2		
**Having PE**	18	19		

**Table:** Table2 Mean Change Scores of Variables As a Function of Time × Group

**Variable**	**Tamarind Group**		**Sertraline Group**		**Statistics (ANOVA)**
	Pretest M (SD) [95% CI]	Posttest M (SD) [95% CI]	Pretest M (SD) [95% CI]	Posttest M (SD) [95% CI]	F, p, ηp²
**PEDT**	14.80 (0.63) [13.52-16.08]	9.85 (0.67) [8.48-11.22]	15.71 (0.62) [14.46-16.96]	9.14 (0.66) [7.80-10.48]	F=126.84, p=.001, ηp²=.76
**Erectile Function **	19.95 (1.26) [17.39-22.50]	23.45 (0.90) [21.63-25.27]	18.05 (1.23) [15.55-20.54]	21.33 (0.88) [19.55-23.11]	F=36.26, p=.001, ηp²=.48
**Organic Function**	7.10 (0.55) [5.99-8.20]	7.70 (0.43) [6.83-8.56]	6.85 (0.53) [5.78-7.93]	7.62 (0.42) [6.77-8.46]	F=9.67, p=.003, ηp²=.20
**Sexual Desire **	6.85 (0.39) [6.05-7.65]	7.60 (0.27) [7.05-8.15]	5.95 (0.38) [5.17-6.73]	7.14 (0.26) [6.61-7.68]	F=32.01, p=.001, ηp²=.45
**Intercourse Satisfaction**	8.90 (0.55) [7.78-10.02]	10.40 (0.48) [9.42-11.37]	7.90 (0.54) [6.81-8.99]	9.24 (0.47) [8.29-10.19]	F=27.73, p=.001, ηp²=.41
**Overall Satisfaction **	7.05 (0.48) [6.07-8.02]	8.05 (0.31) [7.42-8.68]	6.05 (0.47) [5.09-7.00]	7.52 (0.30) [6.90-8.14]	F=47.81, p=.001, ηp²=.55

This analysis was conducted using data from 41 participants. The results indicated that the distribution of the data was normal.

Nor The two groups did not differ significantly in patients’age, t (39)=0.56, P=0.57; the age of the patients’ wife, t (39)=0.72, P=0.48; patients’ education , x2 (6, N=41)=4.63, P=0.59; the education of the patients’ wife , x2 (6, N=41) = 8.06, P=0.23. Moreover, the two groups did not differ significantly on baseline PEDT, x2 (1, N=41)=0.003, P=0.95. Mean scores of the demographic information and statistical variable at baseline are presented in Table-1.

All 41 randomized participants (20 tamarind, 21 sertraline) had complete data available at the 4-week follow-up and were included in the analysis. Scores of the PEDT and subscales of IIET were submitted to a 2 (Group: Tamarind- Sertraline) × 2 (Time: pretest, posttest) mixed repeated measures ANOVA. Results showed that the main effect of the time was significant, F (6, 34)=22.79, P=0.001, ηp2=0.80, in terms of all variables including PEDT, F (1, 39)=126.84, P=0.001, ηp2=0.76, Erectile Function, F (1, 39)=36.26, P=0.001, ηp2=0.48, Orgasmic Function, F (1, 39)=9.67, P=0.003, ηp2=0.20, Sexual Desire, F (1, 39)=32.01, P=0.001, ηp2=0.45, Intercourse Satisfaction, F (1, 39)=27.73, P=0.001, ηp2=0.41, and Overall Satisfaction, F (1, 39)=47.81, P=0.001, ηp2=0.55. This shows that the pretest scores of patients differ significantly from the posttest scores in both groups. However, the main effects of Group, F (6,34)=0.64, P=0.69, ηp2=0.10, and the interaction between Time and Group, F (6,34) =1.06, P=0.42, ηp2=0.15; were not significant for any of the studied variables (see Table-2 for mean change scores). These results suggest that both treatments were equally efficient in treating PE. Side effects were minimal in both groups. In the tamarind group, a small number of participants reported mild symptoms, such as gastrointestinal discomfort, while in the sertraline group, a few participants reported mild symptoms, such as nausea or headache. Exact numbers and specific side effects were not systematically recorded due to their mild nature. No severe adverse events were observed. No ancillary analyses, such as subgroup or exploratory analyses, were conducted.

## Discussion

Various treatments are currently available in medicine and behavioral therapy to improve premature ejaculation. Traditional medical approaches often involve standard medications, such as SSRIs. However, recent research has also explored newer options, including topical medications and herbal remedies. Given the growing interest in medicinal plants and the need for thorough scientific investigations into their efficacy, this study was designed to compare the effects of tamarind and sertraline on premature ejaculation.

In this study, 20 subjects were assigned to the tamarind group and 21 to the sertraline group. After ensuring that both groups were balanced in terms of functional factors, the results of the intervention revealed no significant differences between the groups based on the criteria from the IIEF and PEDT questionnaires.

Quantitative analysis revealed a significant difference in questionnaire scores in both groups. Previous studies have shown that several drugs in the SSRI category have been approved for treating premature ejaculation, with sertraline being recognized as a standard treatment [11]. Some studies indicate that higher doses of sertraline may be more tolerable and effective for treating premature ejaculation compared to more commonly used treatments, such as Dapoxetine [26][27]. There have not been many studies on the effects of tamarind on premature ejaculation. The study mostly relevant to ours was conducted by Homayounfar et al., [16] which had a distinct advantage due to the inclusion of a placebo group. However, our current study is preferable because of the precise homogenization of the participants. In the Homayounfar’s study, [16] the ejaculation time and the PEDT score in the paroxetine group showed significant improvement compared to the tamarind and placebo groups. In contrast, our study did not measure ejaculation time due to a lack of cooperation of the patients. Nevertheless, we found that the PEDT questionnaire scores improved significantly in both groups following the intervention, although there was no significant difference between the two groups after the intervention. Additionally, the scores for orgasmic function and intercourse satisfaction in the paroxetine group significantly increased compared to the other two groups. However, our results were different in terms of orgasmic function and intercourse satisfaction as the effect of tamarind on these factors was significant and did not differ meaningfully from the sertraline group.

One noteworthy aspect of the present study was the use of tamarind at a dosage of 80 mg, which differs from the 130 ml of tamarind powder combined with 260 mg of sugar (totaling 360 mg) used in the Homayuonfar’s study [16]. This discrepancy may be attributed to differing responses to treatment at different sites. Some studies suggest that in patient groups with controlled and uncontrolled sugar levels, the combination of sugars—often utilized as excipients in pharmaceutical formulations—can influence pharmacological effects [28]. These effects may include alterations in drug absorption rates and potential drug degradation [29], ultimately contributing to a reduction in drug consumption. Additionally, the present study emphasized the importance of educating the patient's spouse based on the findings, an aspect that was not addressed in the research conducted by Homayunfar et al. [16].

Despite the limitations of various studies, it has been observed that the tamarind plant contains compounds such as flavonoids [30], antioxidants [31], and anti-inflammatories [32] in its different parts. This is significant because research has indicated that these properties of tamarind may help improve premature ejaculation in certain contexts. For example, animal studies [33] have shown that flavonoids can enhance sexual function. Additionally, tamarind has been linked to improved blood oxidative levels in patients undergoing treatment for premature ejaculation with SSRI drugs [34], and inflammation [35] has been identified as a factor in the occurrence of premature ejaculation. Despite the proven benefits of tamarind, one study found that this plant's products can have toxic and teratogenic effects on the embryos of zebrafish [36]. Both treatments exhibited minimal side effects, supporting their safety for short-term use in treating premature ejaculation.

Although our research yielded significant findings, several limitations should be addressed in future studies. The first limitation is the small sample size, which may reduce the statistical power of the study and restrict the generalizability of our results. The second concern is the cross-sectional nature of the study and the absence of follow-up sessions, raising questions about the long-term stability of the findings. The third issue refers to the lack of a control/placebo group, which makes it challenging to distinguish between psychosocial effects, such as the placebo effect, and the actual effects of the treatments.

The last limitation refers to ignoring the cultural and social diversity of subjects; if participants are selected from a specific geographical or cultural region, their perspectives on the topics included in the questionnaire may be biased. Addressing the mentioned issues in future research could enhance the reliability of the results. Furthermore, future research is recommended to explore the specific mechanisms behind the effects of tamarind on sexual function through both laboratory and clinical methods. It is also important to examine the long-term safety of tamarind use and to compare its effectiveness with other common treatments for premature ejaculation.

## Conclusion

This study is the first to investigate the effects of tamarind seed extract (Tamarindus indica L.) in treating premature ejaculation, comparing it to the well-known treatment Sertraline through a randomized double-blind trial. Participants in our study were randomly assigned to different groups, which helps reduce bias and enhances the internal validity of the findings. Furthermore, this study assessed various aspects of sexual function, including erectile function, orgasmic function, sexual desire, intercourse satisfaction, and overall satisfaction, in addition to premature ejaculation. While the results indicated positive effects from the Sertraline intervention, the tamarind extract also demonstrated similar benefits in improving premature ejaculation and other related factors. The findings of our study can serve as a foundation for alternative treatments for individuals experiencing premature ejaculation.

## Conflict of Interest

None declared.
